# Progress of Veterinary Science

**Published:** 1881-10

**Authors:** 


### PROGRESS OF VETERINARY SCIENCE.

---

Fouquet's Method of Producing Either Sex at Will, of which there was much talk a year ago, has, it is said, been further tried and with good results.

The method consists in feeding up the cow, while the bull is put on low diet, and his sexual powers weakened by previous constant exercise of them.

A new Parasite in Pork.—J. Duncker, of Berlin, has discovered a new parasite in pork. He describes it as a small leech-like worm, of a hitherto undescribed species. The animals are found moving about very actively among the muscular fibres and the connective tissue investments. The "Zeitschrift fuer mikroscopische Fleischbeschan und populaere Mikroskopie," contains a full description of them, accompanied by illustrations.—*Chicago Medical Review.*

Lampas.—Many horses fall off in flesh and refuse to eat from the above trouble. Until this trouble is recognized, the horse suffers and fails to respond to the effects of various tonics, etc., administered to restore his failing appetite. If the mouth is examined, when lampas exists it will be found that the gum has grown down even with the upper front incisors, and often ahead of them. The remedy is to cut out the redundant growth, or burn it down with a hot iron. Some experienced horsemen say that this may be remedied by feeding on corn in the ear.—*Southern Clinic.*

Malaria in Horses.—Dr. Moore (*Country Gentleman*) believes that horses may be subject to that aerial poisoning, and even die from it. In answer to a correspondent, he says: "While I was free to state at first that I could not say positively what caused their death, yet I think that later investigation will prove it to be malarial fever, and paralysis of various organs as a consequence. The throat was paralyzed, and later the limbs and tail were partially or wholly so." He then relates a case in his own practice of what seemed to be malarial poisoning.

Treatment of Chicken Cholera.—The remedy used by Dr. J. W. Cooper is:

Cayenne pepper, two parts; prepared chalk, two parts; pulverized gentian, one part; pulverized charcoal, one part—all by *measurement* and not by weight. Mix all well together, and form a paste with either lard or mutton suet; and, for a fowl already diseased, give a piece the size of a common marble, once a day, and keep in a warm, dry place 48 hours, when a cure will be effected. As a preventive, make a paste of cayenne pepper, one part; prepared chalk, one part; pulverized gentian, two parts—by measurement as before. If the disease be in the neighborhood, give, once a week, an ordinary sized pill to grown fowls—smaller birds in proportion.

Of this treatment the essential thing is the cayenne pepper. Its utility is well established.

A MOST INSTRUCTIVE ARTICLE ON THE HORSE will be found in the last edition of the *British Encyclopædia.* The anatomy of the horse (written by Prof. Flowers) has received an impetus since Huxley took it as the theme for his celebrated lectures on evolution. The history and management of the animal are treated of by E. D. Brickwood, and the racehorse is described in an article by E. D. Brickwood, Major J. R. Hubbard and W. T. Chester. "American Horses," writes Mr. Brickwood, "have sometimes been sent over to compete in England, but, on the whole, they have not been very successful, as they have found the English horses too good for them."

A NEW HORSE.—M. Poliakoff, the distinguishsd Russian naturalist, has examined a horse presented by Colonel Prejvalsky to the St. Petersburg Academy, and decides it to be a new species, which he has named *Equus Prejwalskii.* It appears that the new representative of the family of undivided-hoofed mammals is in some respects intermediate between the domestic horse and the wild ass, but it differs from the asinine genus in having four callosities, one on each leg. In the form of skull, absence of dorsal stripe, and other particulars, it resembles the domestic horse. This newly-recorded animal is indigenous to the plains and deserts of Central Asia, and has not hitherto fallen under the dominion of man.

THREE FOALS AT A BIRTH.—In March last an English mare, 21 years years old, gave birth to three foals. The first came dead, the other two alive. A couple of years previous to this, the same mare brought forth twins. The latter, even, was extraordinary for a mare, but triplets are much more so. Cows often produce twins, and occasionally triplets, or a greater number. Not long since we heard of a cow dropping *seven* calves during the day, all of which were alive. We should like to know the weight of these at their birth, and their subsequent history. Sheep often produce twins and triplets, and a greater number of lambs are not uncommon. Sows generally drop from seven to ten pigs, and now and then we hear of this number being doubled, or even this exceeded. Twenty-four live pigs at a birth is the maximum within our recollection.—*Live Stock Journal.*

HOW TO KILL ANIMALS WITH A BLOW.—The end to be reached by a blow upon the head is the derangement of the brain, which causes loss of consciousness, and therefore insensibility to pain. Many persons strike too high, and fail in bringing the animal to the ground—not from a too feeble blow, but a wrong application of it to the head. The point to be aimed at in horses is the point of intersection of the lines joining the base of the ear with the opposite eye. There is little or no brain "between the eyes," and this is too frequently the point struck by the axe. With cattle, the brain is nearest the surface, and most readily reached by a blow given on a point which is the intersection of lines drawn from the base of each horn to the eye on the opposite side of the face. In all cases when a blow is administered, the head of the animal should be securely fastened, and as a precaution against any jerking of the head, it is well to blindfold the animal.—*Country Gentleman.*

INTESTINAL CONCRETIONS IN THE MONKEY.—The Bezoar or guliga stones are concretions or calculi formed in the "stomach and intestines" of the red monkey, a species of Semnopithecus abundant in the interior districts of Borneo. Mr. A. H. Everett says (in the *Ann. and Mag. Nat. Hist.*, March), that "accounts vary very much among the natives as to the exact position in which the guligas are found, some saying they may occur in any part of the body, others that they occur only in the stomach and intestines, whilst I have heard others declare that they have taken them from the head and even the hand." (Everett does not state what would seem more probable, that they may be renal calculi.) The monkeys drink the water of certain springs, which must be saline mineral springs, or the creatures are cut off from the use of fresh water. Everett adds that "the widespread idea of the medicinal virtue of these concretions would lead us to suppose that there is some foundation for their reputation."

Splenic Fever in the Dog.—A very complete account of this somewhat rare affection is given by H. Engel, in *Wochenschrift fur Thierheilkunde und Viehzucht*, July, 1881. After giving the literature of the subject, which is very slight in amount, he relates the case of a shepherd's dog which ate the flesh of a lamb that had died of splenic fever. One evening soon after this, the dog showed signs of restlessness; he barked and whined all night, and did not eat. In the morning he had a fever, with a painful swelling upon the back. He died the same day. Two other dogs caught the disease from this one. They were taken at once to the dog hospital, and were found to have a temperature of almost 108°, and a pulse of 100. They were treated with a mixture containing half an ounce of brandy and ten grains of carbolic acid, internally, and the carbuncles were frequently washed with carbolic water. But the patients soon died.

The Dog and Tape-Worm in Iceland.—Some serious facts concerning the above are recorded by Mr. St. George Mivart in a late number of the *Contemporary Review*: "The egg of a certain tape-worm may be voided by some dog into a stream or rivulet, and if such egg be accidently drank in by a human being, it grows into a kind of bladder-worm, the ravages caused by the growth of which are so serious that in Iceland (where the social conditions lead to the maintenance of many dogs) it is estimated to be the cause of one death out of every seven. So great is the evil that the Iceland Legislature, some years ago, ordered that all the dogs of the island should be simultaneously purged and their excreta burned. These facts with respect to the dog show that the filthy unclean habit in which so many ladies indulge of allowing their lap-dogs to lick their hands and faces, is a practice not unattended with danger."

The Distance Animals can Swim.—Animals themselves are capable of swimming immense distances, although unable to rest by the way. A dog recently swam thirty miles in America in order to rejoin his master. A mule and a dog washed overboard during a gale in the Bay of Biscay have been known to make their way to shore. A dog swam ashore with a letter in his mouth at the Cape of Good Hope. The crew of the ship to which the dog belonged all perished, which they need not have done had they only ventured to tread water as the dog did. As a certain ship was laboring heavily in the trough of the sea, it was found needful, in order to lighten the vessel, to throw some troop-horses overboard, which had been taken in at Corunna. The poor things, my informant, a staff-surgeon, told me, when they found themselves abandoned, faced round and swam for miles after the vessel. A man on the east coast of Lincolnshire saved quite a number of lives by swimming out on horseback to vessels in distress. He commonly rode an old gray mare, but, when the mare was not to hand, he took the first horse that offered.—*Popular Science Monthly.*

Death from Rupture of the Rectum Occasioned During Attempts at Copulation.—The following case is reported by Dr. A. A. Holcombe in the *Vet. Review:*

I have just lost a 14½-hand six-year-old brown mare that was sent to a sixteen-hands-high stallion to be covered. A young man about 18 years old was in charge of the stallion, and during the attempt to effect copulation the penis entered the rectum and lacerated the left wall to the extent of six inches or more. The wound through the organ was situated about eight inches from the anus, and extended some six inches into the surrounding tissues. But slight hæmorrhage resulted. Colicky pains came on in a short time, and lasted until death. The fœces gained access to the wound and served to increase the irritation and suffering. Excessive effusion followed, during the course of twelve hours. Peritonitis set in, and at the end of 36 hours the patient was destroyed, as it was believed impossible that recovery could take place. No post-mortem examination was made.

How Ostriches Digest.—A correspondent of the *Pharmaceut. Journal*, writing from the Cape of Good Hope, where the ostrich is domesticated, states that he is perfectly certain that the digestive powers of the ostrich are very small, and only effective on certain kinds of food, and that the ten-penny nails and iron bolts upon which its digestive reputation is based, are not digested at all, but simply bent and crushed by a powerful gizzard that is so thick and tough as to be with difficulty cut by a knife. The writer vouches that he has himself seen buttons and coins that have been worn to the thinness of wafers by the grinding action of this organ, and that the hardest stones assume a fine polish under its action. It would be a pity to spoil, by a paraphrase, the *naïvete*, of what follows: "When a bird is in poor condition, and fed on maize, by carefully dividing the breast feathers, you can hear this gizzard, or living grinding-mill, at work." Presuming upon the possession of such an apparatus, ostriches eat so enormously that stoppages, which, unless relieved, will kill a bird in twenty-four hours, not unfrequently result; powerful drastics are then given, of which they are said to require a larger dose than a horse.

The Blood in Hibernation.—In the autumn of 1879, Professor V. Wittich received twelve living German moles (*Mus montanus*), in order to investigate the amount of glycogen in the liver during their winter sleep. One animal was killed accidentally immediately after its arrival, and the results obtained on a microscopical examination of the blood, led to an examination of the blood of the other animals, all killed in perfect health, and in all the same appearances were found. One of the animals had died on the journey without signs of disease, although perhaps in consequence of a bite from another animal.

The blood taken from a vein in the peritoneal cavity contained a large number of thread-like moving objects, which moved the blood corpuscles lying near them, and, after diluting the blood with a-half per cent. solution of chloride of sodium, presented an appearance identical with that of the spermatozoa of the frog. These organisms appear to be the same as those described by T. R. Lewis, as existing in the blood of healthy rats. They are much larger than the spirilla met with in the blood of relapsing fever They were found in the blood from various parts, arterial and venous, in every animal. They disappear from the blood as soon as decomposition sets in and the putrefaction-bacteria appear. They were very numerous, ten or twelve being found in every drop of blood, and they furnish a new proof of how large a number of parasitic organisms may exist in the blood without causing any disturbance to the general health. Attempts were made to inoculate guinea-pigs, but in every instance with a negative result. —*London Lancet.*

Upon the Presence of the Virus of Rabies in the Cerebral Substance of Mad Animals.—Mr. Pasteur has presented recently at the Academy of Medicine of Paris a note upon the presence of the rabid virus in the brain substance of rabid animals. Since the fact published by Meynert, in 1869, that nervous lesions were found in the spinal marrow of two children which had died with hydrophobia, similar facts were reported by many pathologists in Germany, France and America, which demonstrated the presence of lesions of the nervous centres amongst those of rabies. Mr. Pasteur has had the idea of hunting the virus in the substance of nervous centres in animals which had died of that disease. His experiments, assisted by M. Chamberlain, Roux and Thuilier, were successful. The rabid virus exists in the substance of the nervous centres with a power of action as great as in the saliva. More than that, in inoculating the cerebral substance of an animal dead by hydrophobia, in the brain of a trephined dog, Mr. Pasteur has succeeded in shortening the duration of the inoculation, which thus does not last more than a week. This will facilitate the experimental researches upon this disease, which is yet so mysterious.

The Turcoman Horse.—A great deal has been said and written about Turcoman horses, I am fain to believe, by people who make their statements from mere hearsay, who never had an opportunity of judging personally of the wonderful qualities they so freely announce. During my present stay at Merv, and a pretty long one among the Yomuds, I have had ample opportunities of judging of the powers of Turcoman horses, and of hearing their praises sung by no cold partisans of the breed—Turcomans themselves. Yet I never witnessed, or even heard of, among the Turcomans, such exploits as were lately printed in a Russian book of travel. At the moment of writing I have Turcomans at my elbow (alas! only too many of them), and I have over and over again made searching inquiries about the power of Central Asian steeds.

A first-class Turcoman horse, after a month's special training, and with ample and special food, will do from sixty to seventy mil s a day and keep it up for an apparently unlimited period. It is this sustained power which is probably their only excellence which has not been overrated. For speed they could not hold their own against the higher class European horses. As a rule the Turcoman horse is very "leggy," but extremely graceful of limb. His chest is narrow but long. His head is handsome; but the neck at the point of junction has a strangled appearance. The neck, too, far from having the proud curve of an Arab horse, is not even straight. It is slightly concave from above, which gives to some otherwise gazelle-looking animals a lamentable resemblance to a strangely abnormal camel.—*English Correspondence.*

String-Halt.—This peculiar snatching up of the hind leg is no doubt produced generally by an affection of the hock. The extensor-pedis seems to be the muscle most severely affected, though not the only one, however, which is disturbed and thrown into spasmodic action. The producing causes may be accurately attributed and confined to two in number, the loss of nervous influence and volition, and the peculiar construction and articulation of the hock-joint. If the leg of a horse be stripped after death of its muscles and the ligaments of the limb remain undisturbed, and if the limb both above and below the hock-joint be mechanically straightened out by the hands of the operator, the moment he lets the limb loose from his grasp it will immediately spring back again to a bent position. Thus an imitation of string-halt is produced, the balance of power not being equally proportioned in the limb. The articulary ligaments of the hock-joint are much stronger than the muscles of the thigh; consequently the moment the horse lifts his foot to advance or move it forward under his body the limb is spasmodically snatched up by the power of the ligaments. Whether this explanation be well founded or otherwise, the fact is well established in my mind from practical experience and close observation made on this spasmodic affection of the muscles that the seat of the difficulty is in the hock-joint. I have noticed recently published accounts by some of the knowing veterinary surgeons that string-halt can be cured by blistering. I have yet to see a single cure that ever has been accomplished by blistering or any other medical treatment. I have no hesitancy in asserting that nò treatment is of the slightest avail. Horses afflicted with string-halt are just as qualified and able to perform all kinds of work or driving as those not affected with it, and in many instances the spasmodic action of the ligaments does not increase but remains in the same condition as when the attack first developed itself.—Dr. E. S. Smith, in *New York World.*

Prevention and Treatment of Milk Fever.—One of the best methods of preventing milk fever, is to feed the cow, several weeks to several months before calving, according to its danger—if in winter, on ordinary dry hay only, with a quart or so of wheat bran, night and morning, to keep the bowels open; if in summer, let her run on a poor pasture, and at all times have a large lump of Liverpool rock salt, to lick at pleasure. If the cow has been dried off a couple of months before due to calve, watch the approach of parturition, and if the bag shows extra full, then begin to draw a small quantity of milk from it two weeks or less before her time, and increase this, according to the fulness of the bag, till the calf is dropped; then milk her clean after the calf has sucked, at three equal intervals of every twenty-four hours. In the meanwhile, do not increase her feed for a month or more, till all danger of fever is passed. If the cow has continued to give milk up to within a few days of the time for her to calve, as is sometimes the case, then perhaps it will not be necessary to milk her till after calving. Keep her dry and sheltered from storms and from excessive cold or heat. See that the water she drinks is pure, and that she has all she wishes to take, at least three times per day. Never let this water get icy cold, and after calving, give it slightly warm for a few days.

As soon as affected, if not already in a comfortable stable, put the cow into one; litter the floor well, and always keep this dry and clean. One of the most simple and effectual prescriptions for this disease is half a pound of Epsom salts, dissolved in three or four quarts of warm water, mixed with two table spoonfuls of sweet spirits of nitre. Wet up a small feed of wheat bran with this. If the cow will not take it so, then put the salts and nitre solution into a strong-necked bottle, trice up her head and pour it down the throat. Repeat this every morning till cured. This simple remedy rarely fails, even in the worst cases, if all the above directions are carefully followed. Rub the bag with lard, mixed with the last strippings, every time the cow is milked. This renders the bag soft and pliable, and prevents maminitis.—*National Live Stock Journal.*

PASTEUR'S EXPERIMENTS IN VACCINATING ANIMALS.—When Pasteur discovered a method of so cultivating the specific germs of chicken cholera as to give them the power of preventing the disease when inoculated, he asserted it to be possible to treat other specific organisms in the same way. This he has recently done for the virus of charbon. He was furnished with an unusually good opportunity of testing the power of his modified virus. The Agricultural Society of Melun put at his disposal sixty sheep, to be experimented with as he chose.

Ten of these were separated from the rest and kept subject to inspection. Twenty-five others were inoculated twice with the cultivated virus of charbon. After a sufficient time these and another twenty-five sheep were inoculated with the pure virus of the disease. The fifty sheep were then allowed to mingle together in the same inclosure. The twenty-five not "vaccinated" did not take the disease, while the others did. Those of the latter that died were buried in an inclosure together. A certain number of vaccinated and unvaccinated sheep were then turned into this inclosure and allowed to pasture there. The unprotected were soon attacked with the charbon, while the others were not. Thus M. Pasteur claims to have demonstrated not only the protective power of the modified virus, but also a previons assertion, that sheep pasturing upon ground where animals killed with charbon are buried, will catch the disease.

M. Pasteur made similar experiments with ten animals of the bovine species, and asserts that the same protective power was secured to them.

Pasteur's communication was received with applause by the society before which it was read, yet it was not allowed to pass without criticism. M. Colin claimed, and with justness, that similar results had been obtained by himself a year ago, and by Toussaint a little later. He might have added that Greenfield and Burdon-Sanderson had also experimented successfully in the same line. Still no one has ever obtained successful results upon so large a scale or with so much uniformity.

It was also suggested that it yet remains to be proved that the preventive power of the inoculations lasts for any considerable length of time.

Whatever be the question of priority, however, it is certain that Pasteur's results have attracted the most attention of any yet made in this line. And they will probably give a fresh impulse to studies in this direction. It must be remembered that the successes in preventive inoculation have, so far, been with diseases having a close pathological resemblance. And before the experiments of Pasteur, their practical application did not seem easy.

At the meeting of the International Medical Congress in London, last August, Pasteur reviewed his experiments, and renewed more strongly than ever his claims. A full account of his paper is given in the New York *Medical Record.*

CHICKEN CHOLERA.—A very intelligent account of this disease, of which so much has lately been said by M. Pasteur, has been given by Dr. S. J. Parker, in the *Country Gentleman.*

This disease begins by indigestion. The infected fowl eats heartily and drinks excessively, from a morbid appetite, but with a paralysis of digestion, so that at night it goes to bed with its crop enormously distended. It may several times, by a diarrhœa, disgorge this load; but usually it slowly gets off of the roost with its still distended crop. It may eat again voraciously, but usually is dull and sluggish. The outer edges of the combs are now purplish, but the base of the comb has the natural color. In this stage the fowl can be saved. I find it best to force down its throat *Eucalyptus globulus*, ten drops of the strong tincture; common salts, four to six grains; and half a teaspoonful of ground cayenne (red) pepper. One dose, in a tablespoonful of water, is given at once. If the dose takes effect, digestion is resumed, and in twenty-four hours the fowl is relieved, or decidedly better.

If the disease goes on unchecked, the next symptom is costiveness, with a high fever. The fever usually assumes the type of all critical fevers in the human subject; that is, the hen may seem comfortable, or cold, or with shivers, until nine or ten o'clock A.M., when the fever comes on, with panting breath, legs hot to the hand, and body very hot; comb purple, skin at first a little purple, which becomes on the second, third, or fourth day almost black with the heat of the fever. At four or five in the afternoon the fever recedes, and for an hour or two the fowl may be lively, feed, and seem as if better until it goes to roost. But each day the fever is worse, until, generally in the night, the loaded crop and bowels are emptied by a free discharge of dirty, blackish-green matter, with whitish matter stained yellow. If this discharge is sudden and profuse, the fowl soon after becomes helpless and dies, with a few kicks, croakings, and convulsions. If, however, there is a diarrhœa that keeps the crop and bowels free, there may be the same kind of discharges, which may be small in quantity and not oftener than in health; the blackish-green, the fluid like starch water, and the white overlaid with bright yellow being the only marks of the discharges that really have any peculiarity differing from the discharges of health which an ordinary person can discover. The marks of this stage of the disease are the purple, dark blood in the combs, the sick appearance of the fowl, the panting breath, and a peculiar rise and fall of the under bill, or mandible of the fowl.

This form of the disease is a lingering one, and the patient only lingers a few days longer, and dies a less painful death. A few, say one out of ten, may not die at all, but, after weeks, may half recover. I say "half recover," because I never saw a recovered bird that made a useful fowl, after there has been any diarrhœa.

THE DANGERS OF THE LIVER ROT IN SHEEP.—In a report on the liver rot by Finlay Dunn, in the Journal of the Royal Agricultural Society, England, we are told that "a succession of wet seasons has spread the liver rot widely through most of the midland, western and southern counties of England." The increase of the average rainfall by one-fourth has cost England, in the one item of sheep sacrificed, no less than 10,000,000 pounds sterling!

There is no telling when the frightful disease may devastate the flocks of the United States. There should, therefore, be an accurate knowledge of its character, and mode of treatment, and prevention among veterinarians. The following concise account, given in the *Country Gentleman*, may be of interest:

"The sheep rot is caused by a small parasitic animal (*Fasciola hepatica*), which flourishes in ducts in, and leading to, the liver; on this account it is called the liver fluke. The animal resembles a leech, it being a sucking worm, of an oval shape, thick and rounded at one end.

"The mature fluke, as found in the sheep, stands at the end of a series of transformations through which the parasite has passed. To begin with the egg, it, after leaving the mature fluke, passes into the intestines of the host, and is thrown off in the excrement of the sheep. If the egg falls upon dry soil it soon perishes, but if moisture is met with, it hatches into a small embryo which moves by minute hair-like appendages. In a short time the young fluke is provided with legs, and in this state it enters into the body of some snail, and there begins the first part of its parasitic life. The fluke soon becomes encysted or enclosed in a kind of sack, within the flesh of the snail. In this state the fluke is taken up by the sheep, which feed upon grass bearing the snails, and when in the stomach of the sheep, the active leech-like form of the parasite is quickly assumed, and then, finding its way to the ducts of the liver, it reaches the last and perfect state of its transformations, and soon does its destructive work. It is now easy to see the relation between the wet season and the prevalence of the fluke the following year. The hatching of eggs is dependent upon an abundance of moisture. The young fluke, after hatching from the egg, must find a snail and enter its flesh, which it will be much more apt to do in a moist season, the distribution of snails being wider and their number greater during a period of rains.

"*Symptoms of the Disease.*—Flukes, to a small number, may be present in sheep and not be suspected. In the first stages of the 'rot' there is an increased appetite, and a tendency to fatten. Some English feeders have exposed their flocks to the fluke to hasten their animals for the butchers. It may be said that the fat is high-colored and, of course, not of the best. Soon after this period of unnatural excitement there comes a falling away in the condition of the animal; the color of the eye becomes 'tallowy,' the skin wet, and the wool loose. At length a watery tumor appears below the lower jaw, after which the sheep soon dies in a state of rottenness.

"*Remedy.*—The treatment must be wholly in prevention, for when the flukes have once found entrance to the sheep's liver in any considerable numbers, there is no removing them. The parasites must be kept from entering the sheep, and as they enter in the flesh of snails, the sheep must be kept from feeding on herbage on which the snails are found. This requires that the sheep be kept from wet meadows and all pools of stagnant water. The prevention of the liver rot in sheep is to a large extent a question of drainage."

